# Effect of hysterectomy on ovarian function: a systematic review and meta-analysis

**DOI:** 10.1186/s13048-023-01117-1

**Published:** 2023-02-09

**Authors:** Yibao Huang, Meng Wu, Chuqing Wu, Qingqing Zhu, Tong Wu, Xiaoran Zhu, Mingfu Wu, Shixuan Wang

**Affiliations:** 1 National Clinical Research Center for Obstetrical and Gynecological Diseases, Department of Obstetrics and Gynecology, Tongji Hospital, Tongji Medical College, Huazhong University of Science and Technology, Wuhan, 430030 Hubei China; 2 National Clinical Research Center for Obstetrical and Gynecological Diseases, Wuhan, 430030 Hubei China; 3 Key Laboratory of Cancer Invasion and Metastasis, Ministry of Education, Wuhan, 430030 Hubei China

**Keywords:** Hysterectomy, Ovarian function, Leiomyoma, Meta-analysis, Systematic review

## Abstract

**Background:**

Hysterectomy is one of the most frequently gynecologic surgeries performed in premenopausal women. Many premenopausal patients are unwilling to undergo hysterectomy due to the probable decreased ovarian function. The aim of this study is to determine the effect of hysterectomy on ovarian function.

**Methods:**

A meta-analysis has been reported in line with the Preferred Reporting Items for Systematic Reviews and Meta-Analyses (PRISMA) 2020 and the A Measurement Tool to Assess Systematic Reviews (AMSTAR) guidelines. We mainly searched the Embase, PubMed and Web of Science databases for eligible studies. The outcomes were the levels of common indicators of ovarian function, such as anti-müllerian hormone (AMH), follicle stimulating hormone (FSH), inhibin B, estradiol (E2) and luteinizing hormone (LH). The evidence was synthesized using meta-analysis via fixed or random effect model according to heterogeneity. Subgroup analyses were performed to examine the potential sources of heterogeneity.

**Results:**

The 14 included studies were conducted between 1989 and 2021, involving a total of 1,457 premenopausal women with 760 and 697 in the hysterectomy and control group, respectively. We found that hysterectomy damage ovarian function compared to the control group, with lower AMH level [Weighted mean difference (WMD) = -0.56, 95% confidence interval (95% CI): -0.72 to -0.39, *P* = 0.000], higher FSH levels (WMD = 2.96, 95% CI: 1.47 to 4.44, *P* = 0.000), lower inhibin B levels (WMD = -14.34, 95% CI: -24.69 to -3.99, *P* = 0.000) and higher LH levels (WMD = 4.07, 95% CI: 1.78 to 6.37, *P* = 0.000). In addition, E2 levels have a decreasing trend (WMD = -17.13, 95% CI: -35.10 to 0.85, *P* = 0.631) in the hysterectomy group but were not statistically significant.

**Conclusion:**

Hysterectomy has a negative impact on ovarian function, especially in female patients over 40 years old. So, the older patients should closely monitor their ovarian function for early diagnosis and treatment of menopausal symptoms.

**Supplementary Information:**

The online version contains supplementary material available at 10.1186/s13048-023-01117-1.

## Introduction

As a core reproductive organ, the ovary has two unique functions, producing mature oocytes for fertilization and secreting sex hormones to maintain the normal activities of multiple organs [1]. However, ovarian function appears to decline at the earliest stage during a woman’s lifespan, which is regarded as an ageing pacemaker among females [2]. Menopause is the final step of ovarian ageing, which leads to dysfunction of multiple organs, such as vasomotor dysfunction, osteoporosis, cardiovascular disease, and so on [3].

Hysterectomy is one of the most common gynaecologic surgeries performed worldwide for both benign and malignant conditions [4–6]. Notably, uterine leiomyoma is a common female pelvic tumour and is the most common indication for hysterectomy. However, many premenopausal patients are unwilling to undergo hysterectomy; they desire to maintain an intact uterus and worry about decreased ovarian function after hysterectomy. At present, myomectomy is currently the uterus-preserving surgical treatment of choice for non-submucosal uterine leiomyoma. However, there is a risk of recurrence after myomectomy, especially in patients with multiple uterine leiomyomas. The cumulative risk of recurrence was 4.9% at 24 months and 21.4% at 60 months postoperatively [7]. In addition, other uterus-preserving approaches, such as embolization and focused ultrasonography, have been found to have high recurrence rates. Due to the certain recurrence rate of uterus-preserving treatment, nationwide hysterectomy rates are still high among premenopausal women. Although ovarian preservation is increasingly common, studies have found that ovarian failure occurs 4 years earlier than natural menopause after hysterectomy, and the incidence of severe menopausal symptoms is significantly higher among women who underwent hysterectomy than among the general population [8, 9]. In a word, hysterectomy may make female patients vulnerable to premature menopause and adverse health consequences. Therefore, the association of hysterectomy with ovarian function has important public health implications.

Previous studies have revealed conflicting results when examining ovarian function after hysterectomy. In general, hysterectomy can interrupt the ovarian branch of the uterine artery and reduce the ovarian blood supply by 50 ~ 70%, leading to decreased ovarian function [8, 10]. In a cross-sectional study, women with hysterectomy had significantly elevated serum FSH levels and lower ovarian stromal blood flow indices than age-matched healthy women [11]. However, a few studies reported that hormonal levels were not influenced after hysterectomy. For example, no statistically significant differences were detected among the serum AMH, FSH and E2 levels postoperatively when evaluated at 3 months and 1 year time points [12, 13]. In summary, previous studies have shown contradictory results about the effect of hysterectomy on ovarian function in premenopausal women.

The effect of hysterectomy on ovarian function has not been studied in previous meta-analyses. The purpose of this study was to investigate the effect of hysterectomy on ovarian function compared with a control group with intact uterine or uterine blood supply.

## Methods

The work has been reported in line with PRISMA (Preferred Reporting Items for Systematic Reviews and Meta-Analyses) and AMSTAR (Assessing the methodological quality of systematic reviews) Guidelines [14, 15]. The systematic review was registered on PROSPERO (ID: CRD42022339118).

### Eligibility criteria

Studies had to meet the following criteria to be eligible for inclusion: studies that evaluated premenopausal women's ovarian function after ovary-sparing hysterectomy and had a comparison group with intact uterine. Since few studies were included when the control group has the same age distribution, we appropriately relaxed the inclusion criteria of the control group. In the control group, women have a similar age distribution or undergo uterus-preserving treatment with intact uterine blood supply such as myomectomy, levonorgestrel-releasing intrauterine system (LNG-IUS) and ulipristal acetate. Publication date, study design, study location and sample size were not considered as the basis for exclusion.

### Search strategy

According to the PECO framework for research question, the keywords included hysterectomy (E) and ovarian function (O). The Embase, PubMed and Web of Science databases were screened up to June 11th, 2022, along with hand-searching reference lists. Two independent reviewers performed the data extraction, with a third reviewer double-checking the extracted information. References of the reviews and meta-analyses on the topic were searched for additional studies. The search query was: ((((hysterectomy) OR (hysterectomies)) OR (uterectomy)) OR (metrectomy)) AND ((((((((((((((((((ovarian function) OR (ovarian reserve)) OR (AMH)) OR (anti-müllerian hormone)) OR (anti-mullerian hormone)) OR (mullerian inhibiting substance)) OR (müllerian inhibiting substance)) OR (MIS)) OR (FSH)) OR (follicle stimulating hormone)) OR (E2)) OR (estrogen)) OR (estradiol)) OR (LH)) OR (luteinizing hormone)) OR (AFC)) OR (antral follicle count)) OR (Inhibin B)).

### Study selection

After searching and storing the retrieved information separately by databases, duplicate items were retrieved using EndNote software and were removed. Then, the titles and abstracts of the retrieved studies were reviewed in terms of relevance to the main purpose of the study and based on inclusion and exclusion criteria. Finally, the full text of the selected articles was reviewed for inclusion and exclusion criteria.

### Quality assessment

The identical reviewers independently assessed the risk of bias of the 2 included randomized controlled trials (RCTs) using the version 2 of the Cochrane risk-of-bias (RoB 2) tool [16] and quality of 12 observational studies was evaluated using the Newcastle–Ottawa Scale (NOS) checklist [17]. The NOS checklist has 7 dedicated items, each of which gets 1 point except the comparison item which can get a maximum of 2 points. If a study scores less than 5, it means that the risk of bias in that study is high [18].

### Data extraction and synthesis

After screening, selection and evaluation of the quality of selected studies, data were extracted and recorded. The continuous outcome measures between the hysterectomy group and the control group were transformed and expressed as mean and standard deviation (mean ± SD). Weighted mean difference (WMD) and 95% confidence interval (95% CI) were calculated for analysis. Heterogeneity was quantified by I^2^ statistic and Cochran’s Q test; cut-off values of 25%, 50%, and 75% were considered as low, moderate, and high, respectively [19]. A fixed-effect model was chosen to compute outcomes in low heterogeneity; otherwise, a random effect model was adopted. Due to the severe heterogeneity in FSH, E2 and LH, subgroup analysis was used to identify a potential source of heterogeneity. In addition, the sensitivity analysis was used to detect influential publications.

### Publication bias assessment

Publication bias was assessed using the funnel plot, Begg’s and Egger’s tests. If publication bias was detected, the trim-and-fill method was used to estimate its potential impact on the overall effect sizes.

### Statistical analysis

The combination of the results of the selected studies was done quantitatively with STATA-16 software by using “metan”, “metafunnel”, “metaninf”, “metabias” and “metatrim” packages. All tests were two-tailed, and a *p-value* < 0.05 was deemed to be statistically significant.

## Results

Unlike previous studies, this meta-analysis focused on comparing ovarian endocrine function between the hysterectomy and control groups with comprehensive indicators. In addition, subgroup analysis was used to identify potential source of heterogeneity and factors related to decreased ovarian function in these women.

### Study selection

**Fig. 1:**
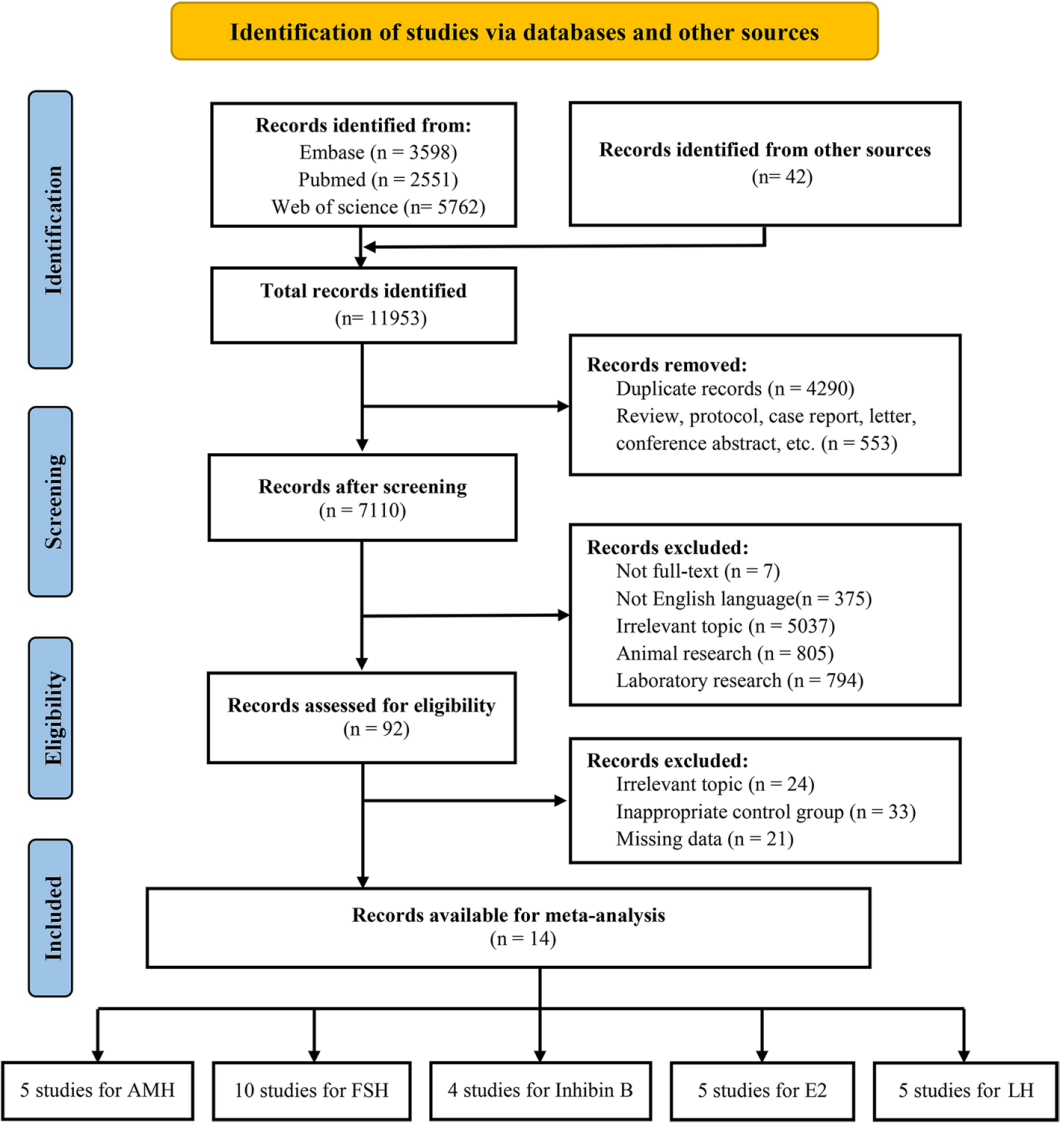
PRISMA flowchart of 14 selected studies

The selection process flow chart is presented in Fig. 1. Initially, 11,911 potential citations were retrieved from the electronic databases, and 42 additional studies were identified by manually searching the reference lists of the included studies. Ultimately, 14 articles were included in the final analysis, including 5 studies examining AMH [20–24], 10 studies examining FSH [11, 22, 24–31], 4 studies examining inhibin B [22, 29, 30, 32], 5 studies examining E2 [22, 28–31] and 5 studies examining LH [24, 28–31].

### Study characteristics and risk of bias

The 14 included studies were conducted between 1989 and 2021, involving a total of 1,457 premenopausal women with 760 and 697 in the hysterectomy and control groups, respectively. Among them, 2 studies were RCTs, 10 studies were cohort studies with a pre-post design (9 prospective cohort studies and 1 retrospective cohort study), and 2 studies were cross-sectional. The sample size ranged from 22 to 320 women. Other detailed information on study characteristics is presented in Table 1 and Table S1.

**Table 1:** Summarized characteristics of 14 selected studies

First author	Year	PMID	Study design	Country	Sample size	Indicators	Evaluation time after surgery	NOS	RoB 2
Atabekoğlu	2012	22,268,398	Prospective cohort study	Turkey	42	AMH	4 m	7	-
Chalmers	2002	12,626,216	Prospective cohort study	Scotland	90	FSH	6 m, 1y, 2y	7	-
Chan	2005	22,268,398	Cross-sectional study	China	30	FSH	43 m (26 m – 53 m)	6	-
Cho	2021	34,300,243	Prospective cohort study	Korea	79	AMH	1w, 2 m, 6 m	6	-
Czuczwar	2018	29,859,107	Prospective cohort study	Poland	53	AMH, FSH, inhibin B, E2	3 m	7	-
Halmesmaki	2007	17,329,045	RCT	Finland	82	Inhibin B	6 m, 1y	-	High
Halmesmäki	2004	1,474,718	RCT	Finland	236	FSH	6 m, 1y	-	High
Hovsepian	2006	16,868,163	Retrospective cohort	USA	22	FSH	1 m, 3 m, 6 m	6	-
Kaiser	1989	2,500,073	Cross-sectional study	Germany	222	FSH,LH,E2	2-10y	5	-
Nahas	2003	12,737,673	Prospective cohort study	Brazil	61	FSH, inhibin B, LH, E2	6 m, 1y	7	-
Qu	2010	20,129,332	Prospective cohort study	China	60	FSH, inhibin B, LH, E2	1w, 3 m, 6 m	6	-
Trabuco	2016	27,054,925	Prospective cohort study	USA	320	AMH	1y	7	-
Wang	2013	24,172,648	Prospective cohort study	China	70	AMH, FSH, LH	2d, 3 m	7	-
Xiangying	2006	16,857,658	Prospective cohort study	China	90	FSH, LH, E2	5d, 1 m, 3 m	7	-

Annotation: *NOS* the Newcastle–Ottawa Scale checklist, *RoB 2* version 2 of the Cochrane risk-of-bias tools, *RCT* randomized controlled trial, *AMH* anti-müllerian hormone, *FSH* follicle stimulating hormone, *E2* estradiol, *LH* luteinizing hormone

The RoB 2 tool was used to evaluate the quality of the 2 included RCTs. Due to the surgical nature, it was difficult to blind study participants and to eliminate deviations from the intended intervention. Thus, all the included trials were deemed to have a high risk of bias for domain of intended interventions (Fig. S1). The quality of the 12 observational studies was assessed by using the NOS checklist. Based on the quality score, all 12 studies scored no less than 5, and no study was excluded (Table S2). The risk of bias summary is presented in Table 1.

### Evaluation of ovarian function in premenopausal women who underwent hysterectomy

#### Serum anti-Müllerian hormone

The meta-analysis included 5 studies that evaluated the association between hysterectomy and serum AMH levels via a fixed effect model. The serum AMH levels in the hysterectomy group were lower than those in the control group (WMD = -0.56, 95% CI: -0.72 to -0.39, *P* = 0.000). In the studies by “Wang (2013)” and “Cho (2021)”, although the baseline AMH levels in the hysterectomy group were lower than those in the control group (Table S3), hysterectomy made this difference more obvious (Fig. S2.a). In addition, there was moderate heterogeneity among the included studies (*I*^*2*^ = 41.3%, *P* = 0.103) (Table 2, Fig. S2.a). Publication bias, checked by using a funnel plot, Begg’s (z = 1.36, *p*-value = 0.174) and Egger’s (t = -2.13, *p*-value = 0.077) tests (Table 3, Fig. S2.b), indicated the low probability of publication bias in overestimating the association between hysterectomy and serum AMH levels. Additionally, the sensitivity analysis generated stable summary estimates without detecting influential publications (Fig. S2.c).

**Table 2:** Summary of meta-analysis results

Indicator	Unit	No. of studies	Model	WMD (95% CI)	*P*-value	I^2^ (%)	P_heterogeneity_
AMH	ng/mL	5	Fixed	-0.56 (-0.72, -0.39)	0.000	41.3	0.103
FSH	IU/L	10	Random	2.96 (1.47, 4.44)	0.000	91.1	0.000
inhibin B	pg/mL	4	Random	-14.34 (-24.69, -3.99)	0.000	77.0	0.000
E2	pg/mL	5	Random	-17.13 (-35.10, 0.85)	0.631	92.8	0.000
LH	IU/L	5	Random	4.07 (1.78, 6.37)	0.000	95.5	0.000

Annotation: *AMH* anti-müllerian hormone, *FSH* follicle stimulating hormone, *E2* estradiol, *LH* luteinizing hormone

**Table 3:** Publication bias of the studies

Indicator	Begg’s test		Egger’s test		Trim-and-fill analyse	
	z	*p*-value	t	*p*-value	Imputed study	Adjusted values
AMH	1.36	0.174	-2.13	0.077	-	-
FSH	1.11	0.267	-0.77	0.450	-	-
inhibin B	1.61	0.108	-2.30	0.061	-	-
E2	2.14	0.033	-2.30	0.042	3	2.83 (-17.37, 23.02)
LH	0.18	0.855	-0.09	0.926	-	-

Annotation: *AMH* anti-müllerian hormone, *FSH* follicle stimulating hormone, *E2* estradiol, *LH* luteinizing hormone

#### Serum follicle stimulating hormone

A total of 10 studies were included in the analysis of FSH levels via a random effect model, and the weighted mean FSH values from the hysterectomy group were higher than those from the control group (WMD = 2.96, 95% CI = 1.47 to 4.44, *P* = 0.000). In the study by “Halmesmäki (2004)”, the baseline FSH levels in the hysterectomy group were lower than those in the control group (Table S3), hysterectomy surprisingly caused the higher postoperative FSH levels in hysterectomy group. Heterogeneity among these studies was high (*I*^*2*^ = 91.1%, *P* = 0.000) (Table 2, Fig. S3.a). Therefore, subgroup analysis was used to identify the potential sources of heterogeneity (Table S4). The results of the subgroup analysis showed that the possible sources of heterogeneity were the patient’s mean age and disease. A mean age ≤ 40 years had less heterogeneity than a mean age > 40 years (*I*^*2*^ = 79.4% vs. *I*^*2*^ = 89.7%, interaction *p*-value = 0.017). In addition, women with menorrhagia had lower *I*^*2*^ levels than those with uterine leiomyoma (28.1% vs. 91.1%, interaction *p*-value = 0.000). Furthermore, no significant publication bias (Begg’s test: z = 1.11, *p*-value = 0.267; Egger’s test: t = -0.77, *p*-value = 0.450) or influential publications were detected among the pooled results (Table 3, Fig. S3.bc).

#### Serum inhibin B

A total of 4 studies evaluating the impact of hysterectomy on inhibin B values were included. As shown in Table 2, the hysterectomy group was found to be associated with significantly lower inhibin B level (WMD = -14.34, 95% CI = -24.69 to -3.99, *P* = 0.000). Significant heterogeneity was detected for these studies (*I*^*2*^ = 77.0%, *P* = 0.000) (Table 2, Fig. S4.a). The results of subgroup analysis showed that world bank countries classification, disease, hysterectomy type, control group are the possible sources of heterogeneity. However, there was only one study in most of these subgroups (Table S5). In addition, no significant publication bias (Begg’s test: z = 1.61, *p*-value = 0.108; Egger’s test: t = -2.30, *p*-value = 0.061) and influential publications were discerned across studies (Table 3, Fig. S4.bc).

#### Serum estradiol

As shown in Table 2, there were no significant differences in E2 levels between the hysterectomy group and control group (WMD = -17.13, 95% CI = -35.10 to 0.85, *P* = 0.631). In addition, there was high heterogeneity among the 5 included studies (*I*^*2*^ = 92.8%, *P* = 0.000) (Table 2, Fig. S5.a). The potential sources of heterogeneity were determined by subgroup analysis, which were mean age, BMI, disease and hysterectomy type. Notably, women with a mean age ≤ 40 years had less heterogeneity than those with a mean age > 40 years (I^2^ = 59.0% vs. I^2^ = 94.7%, interaction p-value = 0.001) (Table S5). Significant publication bias (Begg’s test: z = 2.14, *p*-value = 0.033; Egger’s test: t = -2.30, *p*-value = 0.420) was detected among the included studies (Table 3, Fig. S5.b). Furthermore, the trim-and-fill analyses revealed that publication bias could change the pooled effect size. However, the conclusion (WMD = 2.83, 95% CI = -17.37 to 23.02) remained unchanged after adjusting for publication bias (Table 3). In addition, sensitivity analysis indicated that no individual study had a significant effect on the results (Figure S5.c).

#### Serum luteinizing hormone

The meta-analysis included 5 studies that evaluated the association between hysterectomy and serum LH levels via a random effect model. The weighted mean LH values in the hysterectomy group were higher than those in the control group (WMD = 4.07, 95% CI = 1.78 to 6.37, *P* = 0.000). In the study by “Xiangying (2006)”, the baseline LH levels in the hysterectomy group were higher than those in the control group (Table S3), but hysterectomy also made the difference more obvious (Fig. S6.a). Significant heterogeneity was detected among these studies (I^2^ = 95.5%, *P* = 0.000) (Table 2, Fig. S6.a). Sensitivity analysis showed that the potential sources of heterogeneity were mean age, BMI, disease and hysterectomy type. However, there was only one study in most of these subgroups. Similarly, women with a mean age ≤ 40 years had less heterogeneity than those with a mean age > 40 years (I^2^ = 19.0% vs. I^2^ = 93.2%, interaction p-value = 0.009) (Table S7). In addition, there was no significant publication bias (Begg’s test: z = 0.18, *p*-value = 0.855; Egger’s test: t = -0.09, *p*-value = 0.926), and sensitivity analysis indicated that no individual study had a significant effect on the results (Table 3, Fig. S6.bc).

## Discussion

Previous studies revealed conflicting results when examining the association between hysterectomy and ovarian function. Most of them mainly focused on comparing ovarian function among different hysterectomy surgical approaches without a “healthy” control group [12, 13, 33–36]. This meta-analysis pooled the results from 14 studies with 1,457 premenopausal women. Most baseline hormone levels between the hysterectomy group and control group were not significantly different (Table S3); however, lower serum AMH and inhibin B levels, and higher serum FSH and LH levels were observed in the hysterectomy group after surgery. To our knowledge, this study is the first comprehensive meta-analysis investigating the effect of hysterectomy on ovarian endocrine function compared to a control group with intact uterine or uterine blood supply.

First, hysterectomy damages ovarian reserve compared to the control group. Serum AMH levels strongly are correlated with the number of growing follicles, and therefore, this hormone has received increasing attention as a marker for ovarian reserve [37]. In addition, serum AMH levels remain consistent throughout the menstrual cycle, with no significant variability between the follicular and luteal phases; and therefore this parameter could provide more reliable data on the changes in ovarian reserve after hysterectomy [37–39]. In women younger than 48 years, an AMH value < 0.01 ng/ml had a 51% positive predictive value to predict reaching menopause within 12 months. In addition, a decrease of 0.1 ng/mL in AMH levels increased the risk of early menopause by 14% [40]. In our study, the association between hysterectomy and serum AMH levels was evaluated via a fixed effect model. We found that serum AMH levels in the hysterectomy group were 0.56 ng/mL lower than those in the control group (95% CI: -0.72 to -0.39), suggesting that this surgery could reduce ovarian reserve and cause earlier menopause. Recently, inhibin B has been proven to reflect ovarian reserve effectively and has good consistency with AMH in healthy reproductive women [41]. Our study revealed that serum inhibin B levels in the hysterectomy group were 14.34 pg/mL lower than those in the control group (95% CI: -24.69 to -3.99), which was consistent with the change in AMH levels after hysterectomy. Indeed, hysterectomy disrupts ovarian blood flow and removes paracrine or endocrine signals from the uterus, thereby hastening follicular depletion and leading to earlier menopause [42].

Second, it can be seen from different indicators that hysterectomy damages ovarian function to varying degrees. Generally, the ovarian function indicators consecutively deteriorated as ovarian insufficiency progressed, indicated by an increase in FSH and LH levels but a decrease in E2 levels [43]. Notably, due to postoperative menopause, it is difficult to determine their baseline level in female patients after hysterectomy, and these indicators fluctuate greatly throughout the menstrual cycle, so the pooled results of these indicators have high heterogeneity, especially in E2 levels. FSH is the single hormone used for premature ovarian insufficiency (POI) diagnosis but limited by its high fluctuations during the perimenopausal period [44]. Due to the decreased quantity or quality of follicles, the insufficient secretion of ovarian hormones contributed to a preferential rise in FSH levels through negative feedback. Our findings showed that serum FSH levels from the hysterectomy group were 2.96 IU/L higher than the control group (95% CI: 1.47 to 4.44), which was consistent with the hormone characteristics of ovarian function decline. In addition, we found that serum LH levels from the hysterectomy group were 4.07 IU/L higher than the control group (95% CI: 1.78 to 6.37). Because FSH increased much earlier and more sharply than LH in the pre-POI stage, the FSH/LH ratio significantly increased, which was an independent factor to predict poor ovarian response and associated with poor outcomes in IVF treatment [45]. However, there was limited information about the FSH/LH ratio in these 14 included studies. Moreover, we found that the E2 levels have a decreasing trend (WMD = -17.13, 95% CI: -35.10 to 0.85) in the hysterectomy group but were not statistically significant. Because E2 levels fluctuate greatly throughout the menstrual cycle and baseline levels have great individual differences (Table S3), further reliable data are needed to make a reliable conclusion. The decreased trend in E2 levels may explain the early occurrence and severity of menopausal symptoms after hysterectomy.

Third, the subgroup analyses further showed that decreases in ovarian function after hysterectomy are related to patient age, disease and hysterectomy type (Tables S4, S4, S5, S6). For example, patients older than 40 seemed vulnerable to greater ovarian damage after hysterectomy with higher FSH levels (WMD, 4.28 vs. 1.30), higher LH levels (WMD, 4.84 vs. 1.37) and lower E2 levels (WMD, -35.81 vs. 13.63). Since primordial follicle reserve declines with age, older women are at higher risk of ovarian failure after hysterectomy than younger women. Therefore, older patients should more closely monitor their ovarian function for early diagnosis and treatment of menopausal symptoms.

Despite the important findings in this study, there are also some limitations. First, the study design of the included studies ranged from cross-sectional studies to RCTs, which diminished the quality to some extent. In addition, some studies included in the meta-analysis were conducted by the same author. This is one of the reasons why the heterogeneity was large in most indicators. Although sensitivity analyses showed that excluding studies did not substantially alter the results, these data could reduce the strength of the evidence to some extent. In particular, significant publication bias was detected in E2, which may impact the conclusions of this study. Second, although the sample size was large enough, the total number of included studies was relatively small. There was only one study in most subgroups, which leads to the low accuracy for subgroup analyses in some indicators, such as E2 and LH. Third, some key information of the population in most studies is vague or even unknown, such as BMI, parity, disease and type of hysterectomy. In this situation, meta-regression analysis is not applicable, which may lose some suggestive information for this study.

Despite these limitations, the current study has important implications for clinical practice and policy-making. In clinical practice, many premenopausal patients are unwilling to undergo hysterectomy because they worry about the probable decreased ovarian function after surgery. Our findings revealed that hysterectomy damages ovarian function while the degree is relatively acceptable for surgical indications. This study provides clear evidence for surgeons and patients to facilitate informed decision-making. In addition, the subgroup analyses determined that the decreased ovarian function after hysterectomy was closely related to patient age, disease and hysterectomy type. These variables should be considered as randomization stratification factors in future studies to investigate possible effect modification. In particular, patients older than 40 were vulnerable to greater ovarian damage, which suggested that older patients should be more closely followed up after hysterectomy to check ovarian function for early diagnosis and treatment of menopausal symptoms. In short, our findings suggest that some important questions are worth further exploration, which may contribute to the development of relevant policies.

## Supplementary Information

**Additional file 1:** **Table S1. **Other summarized characteristics of 14selected studies.

**Additional file 2:** **Table S2. **Quality assessment of the 12 included observational studies(1989–2021) based on the NOS (Newcastle-Ottawa Scale) checklist.

**Additional file 3:** **Table S3.** Summary of the baseline hormone levels in patientsbefore hysterectomy.

**Additional file 4:** **Table S4. **Subgroup analysis of FSH.

**Additional file 5:** **Table S5. **Subgroup analysis of inhibin B.(DOC 50 kb)

**Additional file 6:** **Table S6. **Subgroup analysis of E2.

**Additional file 7:** **Table S7.** Subgroup analysis of LH.

**Additional file 8:** **Figure S1.** Bias risk summary for the 2 included RCTs based onthe RoB 2 tools.

**Additional file 9:** **Figure S2.** (a) Forest plot of overall WMD for AMH amongwomen underwent hysterectomy; (b) Funnel plot for assessing publication biaswithin studies related to AMH; (c) Sensitivity analysis for studies related toAMH.

**Additional file 10:** **Figure S3.** (a) Forest plot of overall WMD for FSH amongwomen underwent hysterectomy; (b) Funnel plot for assessing publication biaswithin studies related to FSH; (c) Sensitivity analysis for studies related toFSH.

**Additional file 11:** **Figure S4.** (a) Forest plot of overall WMD for inhibin Bamong women underwent hysterectomy; (b) Funnel plots for assessing publicationbias within studies related to inhibin B; (c) Sensitivity analysis for studiesrelated to inhibin B.

**Additional file 12:** **Figure S5.** (a) Forest plot of overall WMD for E2 amongwomen underwent hysterectomy; (b) Funnel plot for assessing publication biaswithin studies related to E2; (c) Sensitivity analysis for studies related toE2.

**Additional file 13:** **Figure S6.** (a) Forest plot of overall WMD for LH amongwomen underwent hysterectomy; (b) Funnel plot for assessing publication biaswithin studies related to LH; (c) Sensitivity analysis for studies related toLH.

## Data Availability

This systematic review extracted data from publicly available literature. Upon request, the authors are able to share these data with qualified researcher.

## Abbreviations

PRISMA: Preferred Reporting Items for Systematic Reviews and Meta-Analyses
AMSTAR: A Measurement Tool to Assess Systematic Reviews
AMH: Anti-müllerian hormone
FSH: Follicle stimulating hormone
E2: Estradiol
LH: Luteinizing hormone
WMD: Weighted mean difference
95% CI: 95% Confidence interval
RCT: Randomized controlled trial
LNG-IUS: Levonorgestrel-releasing intrauterine system
RoB 2: The version 2 of the Cochrane risk-of-bias tool
NOS: The Newcastle–Ottawa Scale
BMI: Body mass index
