# Supplementary material for: Effect of hysterectomy on ovarian function: a systematic review and meta-analysis
Source: J Ovarian Res. 2023 Feb 9;16:35. doi: 10.1186/s13048-023-01117-1 (PMC9912518; doi:10.1186/s13048-023-01117-1)
Supplement: Supplementary file 1 — Additional file 1: Table S1. Other summarized characteristics of 14selected studies. [file 13048_2023_1117_MOESM1_ESM.doc]

**Table S1.** Other summarized characteristics of 14 selected studies.

| **First author** | **Year** | **Study duration** | **Study group** | **Control group** | **Disease** | **Age**  **(study)** | **BMI**  **(study)** | **Parity**  **(study)** |
| --- | --- | --- | --- | --- | --- | --- | --- | --- |
| Atabekoğlu | 2012 | Unknown | AH | Similar age | Uterine leiomyoma | 44.6 ± 5.6 | 25.2 ± 5.9 | 2.7 ± 1.6 |
| Chalmers | 2002 | Unknown | Hysterectomy | Similar age | Menorrhagia | 36.5 ± 3.3 | Unknown | Unknown |
| Chan | 2005 | Unknown | AH | Similar age | Benign indication | 36.4 ± 11.5 | 22.9 ± 5.0 | 1.4 ± 0.8 |
| Cho | 2021 | 2019 - 2020 | LH | Myomectomy | Uterine leiomyoma | 44.1 ± 3.7 | 24.4 ± 5.3 | 1.6 ± 0.8 |
| Czuczwar | 2018 | 2012.12 - 2013.11 | SH | Ulipristal acetate | Uterine leiomyoma | 39.1 ± 5.5 | 27.5 ± 6.0 | 1.6 ± 0.8 |
| Halmesmaki | 2007 | 1994.11 - 1997.11 | Hysterectomy | LNG-IUS | Menorrhagia | 43.7 ± 3.3 | 25.4 ± 4.5 | 1.9 ± 1.4 |
| Halmesmäki | 2004 | 1994.11 - 1997.11 | Hysterectomy | LNG-IUS | Menorrhagia | 43.1 ± 3.5 | 26.6 ± 5.1 | 2.1 ± 1.2 |
| Hovsepian | 2006 | 2000.4 - 2002.4 | Hysterectomy | Myomectomy | Uterine leiomyoma | 40.1 ± 4.5 | Unknown | Unknown |
| Kaiser | 1989 | Unknown | Hysterectomy | Similar age | Unknown | 38 - 48 | Unknown | Unknown |
| Nahas | 2003 | Unknown | AH | Similar age | Benign indication | 37.6 ± 2.6 | 25.6 ± 3.2 | 2.2 ± 1.4 |
| Qu | 2010 | 2007.8 - 2007.11 | SH | Myomectomy | Uterine leiomyoma | 45.6 ± 3.9 | 24.2 ± 1.9 | 1.0 ± 0.3 |
| Trabuco | 2016 | 2004 - 2007 | Hysterectomy | Similar age | Benign indication | 40.9 ± 4.3 | 31.5 ± 7.3 | 2.0 ± 0.6 |
| Wang | 2013 | 2011.1 - 2011.11 | Hysterectomy | Myomectomy | Benign indication | 41.0 ± 2.1 | Unknown | 1.6 ± 1.1 |
| Xiangying | 2006 | Unknown | Hysterectomy | Myomectomy | Uterine leiomyoma | 40.3 ± 4.4 | Unknown | Unknown |

Annotation: BMI=body mass index; AH=abdominal hysterectomy; LH=laparoscopic hysterectomy; SH=supracervical hysterectomy; LNG-IUS=levonorgestrel-releasing intrauterine system.
