# Supplementary material for: Effect of hysterectomy on ovarian function: a systematic review and meta-analysis
Source: J Ovarian Res. 2023 Feb 9;16:35. doi: 10.1186/s13048-023-01117-1 (PMC9912518; doi:10.1186/s13048-023-01117-1)
Supplement: Supplementary file 2 — Additional file 2: Table S2. Quality assessment of the 12 included observational studies(1989–2021) based on the NOS (Newcastle-Ottawa Scale) checklist. [file 13048_2023_1117_MOESM2_ESM.doc]

| **Author, year, country** | **Selection** | | | |  | **Comparability** |  | **Outcome** | | **Total score** |
| --- | --- | --- | --- | --- | --- | --- | --- | --- | --- | --- |
|  | Representativeness of the sample | Sample size | Non-respondent | Ascertainment of the exposure |  | Confounding factors are controlled |  | Assessment of outcomes | Statistical test |  |
| Atabekoğlu et al., 2012, Turkey | * | - | * | * |  | ** |  | * | * | 7 |
| Chalmers et al., 2002, Scotland | * | - | * | * |  | ** |  | * | * | 7 |
| Chan et al.,2005, China | * | - | * | * |  | * |  | * | * | 6 |
| Cho et al., 2021, Korea | * | - | * | * |  | * |  | * | * | 6 |
| Czuczwar et al., 2018, Poland | * | - | * | * |  | ** |  | * | * | 7 |
| Hovsepian et al., 2006, USA | * | - | * | * |  | * |  | * | * | 6 |
| Kaiser et al., 1989, Germany | - | - | * | * |  | * |  | * | * | 5 |
| Nahas et al., 2003, Brazil | * | - | * | * |  | ** |  | * | * | 7 |
| Qu et al., 2010, China | * | - | * | * |  | * |  | * | * | 6 |
| Trabuco et al., 2016, USA | * | * | * | * |  | * |  | * | * | 7 |
| Wang et al., 2013, China | * | - | * | * |  | ** |  | * | * | 7 |
| Xiangying et al., 2006, China | * | - | * | * |  | ** |  | * | * | 7 |

**Table S2.** Quality assessment of the 12 included observational studies (1989–2021) based on the NOS (Newcastle-Ottawa Scale) checklist.
