# Supplementary material for: Effect of hysterectomy on ovarian function: a systematic review and meta-analysis
Source: J Ovarian Res. 2023 Feb 9;16:35. doi: 10.1186/s13048-023-01117-1 (PMC9912518; doi:10.1186/s13048-023-01117-1)
Supplement: Supplementary file 3 — Additional file 3: Table S3. Summary of the baseline hormone levels in patientsbefore hysterectomy. [file 13048_2023_1117_MOESM3_ESM.doc]

**Table S3.** Summary of the baseline hormone levels in patients before hysterectomy.

Annotation: AMH=anti-müllerian hormone; FSH=follicle stimulating hormone; E2=estradiol; LH=luteinizing hormone.

|  | Study (Year) | Hysterectomy group | | |  | Control group | | | *P* |
| --- | --- | --- | --- | --- | --- | --- | --- | --- | --- |
|  |  | Mean | SD | n |  | Mean | SD | n |  |
| AMH | Atabekoğlu (2012) | 1.46 | 2.02 | 22 |  | 1.53 | 1.82 | 20 | 0.907 |
|  | Wang (2013) | 1.08 | 0.77 | 35 |  | 1.54 | 0.95 | 35 | 0.029 |
|  | Trabuco (2016) | 1.24 | 1.35 | 117 |  | 1.46 | 1.72 | 129 | 0.269 |
|  | Czuczwar (2018) | 2.85 | 1.42 | 26 |  | 3.13 | 1.75 | 27 | 0.526 |
|  | Cho (2021) | 1.10 | 1.72 | 59 |  | 2.40 | 2.72 | 20 | 0.015 |
| FSH | Chalmers (2002) | 4.99 | 4.50 | 55 |  | 5.04 | 2.39 | 31 | 0.954 |
|  | Nahas (2003) | 7.65 | 2.65 | 31 |  | 7.56 | 1.70 | 30 | 0.876 |
|  | Halmesmäki (2004) | 8.40 | 0.60 | 117 |  | 8.70 | 0.80 | 119 | 0.001 |
|  | Hovsepian (2006) | 7.30 | 6.40 | 15 |  | 3.00 | 1.60 | 7 | 0.098 |
|  | Xiangying (2006) | 9.32 | 2.11 | 50 |  | 9.39 | 1.99 | 40 | 0.873 |
|  | Qu (2010) | 12.86 | 10.71 | 30 |  | 12.35 | 13.96 | 30 | 0.874 |
|  | Wang (2013) | 9.30 | 5.03 | 35 |  | 8.30 | 3.06 | 35 | 0.319 |
|  | Czuczwar (2018) | 9.16 | 6.24 | 26 |  | 10.82 | 9.56 | 27 | 0.459 |
| inhibin B | Nahas (2003) | 61.91 | 45.13 | 31 |  | 70.08 | 31.32 | 30 | 0.416 |
|  | Halmesmaki (2007) | 48.62 | 46.34 | 46 |  | 55.10 | 57.07 | 36 | 0.572 |
|  | Qu (2010) | 44.03 | 26.97 | 30 |  | 46.25 | 32.56 | 30 | 0.775 |
|  | Czuczwar (2018) | 28.06 | 11.42 | 26 |  | 29.38 | 25.48 | 27 | 0.810 |
| E2 | Nahas (2003) | 76.49 | 27.95 | 31 |  | 69.64 | 23.02 | 30 | 0.301 |
|  | Xiangying (2006) | 398.0 | 80.21 | 50 |  | 408.61 | 82.66 | 40 | 0.540 |
|  | Qu (2010) | 42.19 | 22.77 | 30 |  | 37.24 | 18.48 | 30 | 0.359 |
|  | Czuczwar (2018) | 88.30 | 27.06 | 26 |  | 102.49 | 45.48 | 27 | 0.176 |
| LH | Nahas (2003) | 3.85 | 1.85 | 31 |  | 4.32 | 1.35 | 30 | 0.263 |
|  | Xiangying (2006) | 10.41 | 1.98 | 50 |  | 9.47 | 2.01 | 40 | 0.029 |
|  | Qu (2010) | 6.99 | 4.30 | 30 |  | 5.82 | 5.12 | 30 | 0.342 |
|  | Wang (2013) | 7.15 | 5.78 | 35 |  | 8.39 | 7.96 | 35 | 0.458 |
