# Supplementary material for: Effect of hysterectomy on ovarian function: a systematic review and meta-analysis
Source: J Ovarian Res. 2023 Feb 9;16:35. doi: 10.1186/s13048-023-01117-1 (PMC9912518; doi:10.1186/s13048-023-01117-1)
Supplement: Supplementary file 4 — Additional file 4: Table S4. Subgroup analysis of FSH. [file 13048_2023_1117_MOESM4_ESM.doc]

**Table S4.** Subgroup analysis of FSH.

| **Potential factors** | | **WMD (CI 95%)** | **No. of study** | **Heterogeneity tau²** | **p-value** | **I^2^** | **Interaction**  **p-value** |
| --- | --- | --- | --- | --- | --- | --- | --- |
| Age | Mean age≤40 years | 1.30 (-0.20, 2.80) | 6 | 4.08 | 0.000 | 79.4% | 0.017 |
|  | Mean age>40 years | 4.28 (2.35, 6.21) | 6 | 9.62 | 0.000 | 89.7% |  |
| Evaluation time after surgery | Short term (≤3 months) | 3.84 (1.32, 6.37) | 5 | 12.23 | 0.000 | 92.1% | 0.258 |
|  | Long term (>3 months) | 2.19 (0.84, 3.54) | 7 | 4.41 | 0.000 | 79.9% |  |
| BMI | Mean BMI≤25 | 3.70 (0.15, 7.25) | 2 | 6.85 | 0.090 | 53.8% | 0.506 |
|  | Mean BMI>25 | 1.59 (-0.77, 3.94) | 3 | 3.65 | 0.066 | 54.5% |  |
|  | Unknown | 3.13 (1.25, 5.01) | 5 | 11.38 | 0.000 | 94.1% |  |
| World Bank countries classification | Upper middle income | 4.49 (2.17, 6.81) | 5 | 10.82 | 0.000 | 90.7% | 0.051 |
|  | High income | 1.77 (0.36, 3.18) | 5 | 4.72 | 0.000 | 81.5% |  |
| Disease | Menorrhagia | 0.37 (-0.61, 1.35) | 2 | 0.35 | 0.234 | 28.1% | 0.000 |
|  | Benign indication | 1.54 ( -0.49, 3.58) | 3 | 2.08 | 0.101 | 51.8% |  |
|  | Uterine leiomyoma | 3.43 (1.00, 5.86) | 4 | 12.28 | 0.000 | 91.1% |  |
|  | Unknown | 4.77 (3.19, 6.35) | 1 | 1.10 | 0.121 | 48.5% |  |
| Hysterectomy type | Hysterectomy (unclassified) | 3.06 (1.32, 4.81) | 6 | 11.04 | 0.000 | 93.3% | 0.112 |
|  | Abdominal hysterectomy | 2.52 (-0.14, 5.19) | 2 | 2.64 | 0.148 | 47.6% |  |
|  | Laparoscopic hysterectomy | 4.31 (-1.48, 10.10) | 1 | 17.71 | 0.041 | 68.6% |  |
|  | Supracervical hysterectomy | -2.67 (-7.10, 1.76) | 1 | 0 | - | - |  |
| Control group | Similar age | 2.64 (1.04, 4.25) | 4 | 4.80 | 0.000 | 84.4% | 0.114 |
|  | LNG-IUS | 2.38 (-1.29, 6.06) | 1 | 3.81 | 0.146 | 52.8% |  |
|  | Myomectomy | 3.55 (1.08, 6.02) | 4 | 13.1 | 0.000 | 92.3% |  |
|  | Ulipristal acetate | -2.67 (-7.10, 1.76) | 1 | 0 | - | - |  |
| All studies | | 2.96 (1.47, 4.44) | 10 | 10.58 | 0.000 | 91.1% | - |

Annotation: BMI=body mass index; LNG-IUS=levonorgestrel-releasing intrauterine system.
