# Supplementary material for: Effect of hysterectomy on ovarian function: a systematic review and meta-analysis
Source: J Ovarian Res. 2023 Feb 9;16:35. doi: 10.1186/s13048-023-01117-1 (PMC9912518; doi:10.1186/s13048-023-01117-1)
Supplement: Supplementary file 5 — Additional file 5: Table S5. Subgroup analysis of inhibin B.(DOC 50 kb) [file 13048_2023_1117_MOESM5_ESM.doc]

**Table S5.** Subgroup analysis of inhibin B.

| **Potential factors** | | | **WMD (CI 95%)** | **No. of study** | **Heterogeneity tau²** | **p-value** | **I^2^** | **Interaction**  **p-value** |
| --- | --- | --- | --- | --- | --- | --- | --- | --- |
| Age | | Mean age≤40 years | -21.06 (-43.29, 1.18) | 2 | 326.76 | 0.001 | 85.5% | 0.435 |
|  |  | Mean age>40 years | -10.83 (-23.64, 1.98) | 2 | 159.05 | 0.003 | 75.1% |  |
| Evaluation time after surgery | | Short term (≤3 months) | -16.43 (-32.95, 0.09) | 2 | 170.71 | 0.006 | 80.6% | 0.779 |
|  |  | Long term (>3 months) | -13.21 (-28.54, 2.13) | 3 | 241.14 | 0.001 | 79.7% |  |
| BMI | | Mean BMI≤25 | -20.27 (-30.19, -10.35) | 1 | 19.63 | 0.261 | 25.5% | 0.302 |
|  |  | Mean BMI>25 | -11.19 (-25.29, 2.92) | 3 | 203.73 | 0.000 | 81.0% |  |
| World bank countries  classification | | Upper middle income | -24.15 (-32.12, -16.19) | 2 | 17.67 | 0.279 | 21.4% | 0.000 |
|  |  | High income | 0.34 (-6.27, 6.95) | 2 | 0 | 0.666 | 0.0% |  |
| Disease | | Benign indication | -31.70 (-44.10, -19.29) | 1 | 0 | 0.617 | 0.0% | 0.000 |
|  |  | Uterine leiomyoma | -14.83 (-27.16, -2.51) | 2 | 110.82 | 0.016 | 71.1% |  |
|  |  | Menorrhagia | 3.01 (-6.28, 12.29) | 1 | 0 | 0.680 | 0.0% |  |
| Hysterectomy type | | Hysterectomy (unclassified) | 3.01 (-6.28, 12.29) | 1 | 0 | 0.680 | 0.0% | 0.000 |
|  |  | Abdominal hysterectomy | -31.70 (-44.10, -19.29) | 1 | 0 | 0.617 | 0.0% |  |
|  |  | Laparoscopic hysterectomy | -20.27 (-30.19, -10.35) | 1 | 19.63 | 0.261 | 25.5% |  |
|  |  | Supracervical hysterectomy | -2.41 (-11.83, 7.01) | 1 | 0 | - | - |  |
| Control group | | Similar age | -31.70 (-44.10, -19.29) | 1 | 0 | 0.617 | 0.0% | 0.000 |
|  |  | Myomectomy | -20.27 (-30.19, -10.35) | 1 | 19.63 | 0.261 | 25.5% |  |
|  |  | LNG-IUS | 3.01 (-6.28, 12.29) | 1 | 0 | 0.680 | 0.0% |  |
|  |  | Ulipristal acetate | -2.41 (-11.83, 7.01) | 1 | 0 | - | - |  |
| All studies |  | | -14.34 (-24.69, -3.99) | 4 | 167.40 | 0.000 | 77.0% | - |

Annotation: BMI=body mass index.; LNG-IUS=levonorgestrel-releasing intrauterine system.
