# Supplementary material for: Effect of hysterectomy on ovarian function: a systematic review and meta-analysis
Source: J Ovarian Res. 2023 Feb 9;16:35. doi: 10.1186/s13048-023-01117-1 (PMC9912518; doi:10.1186/s13048-023-01117-1)
Supplement: Supplementary file 6 — Additional file 6: Table S6. Subgroup analysis of E2. [file 13048_2023_1117_MOESM6_ESM.doc]

**Table S6.** Subgroup analysis of E2.

| **Potential factors** | | | **WMD (CI 95%)** | **No. of study** | **Heterogeneity tau²** | **p-value** | **I^2^** | **Interaction**  **p-value** |
| --- | --- | --- | --- | --- | --- | --- | --- | --- |
| Age | | Mean age≤40 years | 13.63 (0.22, 27.03) | 3 | 133.23 | 0.045 | 59.0% | 0.001 |
|  |  | Mean age>40 years | -35.81 (-61.79, -9.83) | 3 | 1267.67 | 0.000 | 94.7% |  |
| Evaluation time after surgery | | Short term (≤3 months) | -40.39 (-81.59, 0.80) | 3 | 2463.4 | 0.000 | 95.4% | 0.084 |
|  |  | Long term (>3 months) | -0.47 (-19.12, 18.19) | 3 | 552.05 | 0.000 | 89.2% |  |
| BMI | | Mean BMI≤25 | 11.47 (-5.11, 28.05) | 2 | 175.32 | 0.002 | 83.6% | 0.012 |
|  |  | Mean BMI>25 | 11.68 (-11.85, 35.21) | 1 | 308.17 | 0.025 | 73.0% |  |
|  |  | Unknown | -44.81 (-79.49, -10.14) | 2 | 2022.79 | 0.000 | 94.2% |  |
| World Bank countries  classification | | Upper middle income | -19.30 (-43.94, 5.34) | 3 | 912.93 | 0.000 | 94.3% | 0.802 |
|  |  | High income | -14.51 (-42.75, 13.74) | 2 | 1136.30 | 0.000 | 90.0% |  |
| Disease | | Benign indication | 23.68 (11.2, 36.15) | 1 | 0 | 0.000 | 0.0% | 0.001 |
|  |  | Uterine leiomyoma | -33.20 (-61.76, -4.644) | 3 | 1327.06 | 0.000 | 94.5% |  |
|  |  | Unknown | -12.86 (-46.03, 20.31) | 1 | 1046.15 | 0.000 | 92.3% |  |
| Hysterectomy type | | Hysterectomy (unclassified) | -44.81 (-79.49, -10.14) | 2 | 2022.79 | 0.000 | 94.2% | 0.000 |
|  |  | Abdominal hysterectomy | 23.68 (11.22, 36.15) | 1 | 0 | 0.880 | 0.0% |  |
|  |  | Laparoscopic hysterectomy | 11.47 (-5.11, 28.05) | 1 | 175.32 | 0.002 | 83.6% |  |
|  |  | Supracervical hysterectomy | -22.34 (-53.12, 8.44) | 1 | 0 | - | - |  |
| Control group | | Similar age | -0.47 (-24.82, 23.88) | 2 | 833.20 | 0.000 | 90.9% | 0.209 |
|  |  | Myomectomy | -35.11 (-66.72, -3.495) | 2 | 1414.75 | 0.000 | 95.4% |  |
|  |  | Ulipristal acetate | -22.34 (-53.12, 8.44) | 1 | 0 | - | - |  |
| All studies |  | | -17.13 (-35.10, 0.85) | 5 | 966.99 | 0.000 | 92.8% | - |

Annotation: BMI=body mass index.
