# Supplementary material for: Effect of hysterectomy on ovarian function: a systematic review and meta-analysis
Source: J Ovarian Res. 2023 Feb 9;16:35. doi: 10.1186/s13048-023-01117-1 (PMC9912518; doi:10.1186/s13048-023-01117-1)
Supplement: Supplementary file 7 — Additional file 7: Table S7. Subgroup analysis of LH. [file 13048_2023_1117_MOESM7_ESM.doc]

**Table S7.** Subgroup analysis of LH.

| **Potential factors** | | | **WMD (CI 95%)** | **No. of study** | **Heterogeneity tau²** | **p-value** | **I^2^** | **Interaction**  **p-value** |
| --- | --- | --- | --- | --- | --- | --- | --- | --- |
| Age | Mean age≤40 years | | 1.37 (0.42, 2.32) | 2 | 0.18 | 0.291 | 19.0% | 0.009 |
|  | Mean age>40 years | | 4.84 (2.43, 7.24) | 4 | 13.12 | 0.000 | 93.2% |  |
| Evaluation time after surgery | Short term (≤3 months) | | 5.84 (2.5, 9.09) | 3 | 15.01 | 0.000 | 71.8% | 0.064 |
|  | Long term (>3 months) | | 2.49 (1.05, 3.93) | 3 | 2.25 | 0.002 | 71.8% |  |
| BMI | Mean BMI≤25 | | 4.02 (1.96, 6.10) | 1 | 0 | 0.575 | 0.0% | 0.002 |
|  | Mean BMI>25 | | 1.03 (0.26, 1.80) | 1 | 0 | 0.519 | 0.0% |  |
|  | Unknown | | 4.83 (2.10, 7.55) | 3 | 14.11 | 0.000 | 95.0% |  |
| World Bank countries  classification | Upper middle income | | 4.47 (1.32, 7.62) | 4 | 21.74 | 0.000 | 96.9% | 0.640 |
|  | High income | | 3.66 (2.57, 4.74) | 1 | 0.09 | 0.363 | 6.1% |  |
| Disease | Benign indication | | 0.34 (-1.52, 2.20) | 2 | 1.85 | 0.039 | 69.2% | 0.000 |
|  | Uterine leiomyoma | | 7.06 (5.04, 9.07) | 2 | 4.77 | 0.000 | 84.3% |  |
|  | Unknown | | 3.66 (2.59, 4.74) | 1 | 0.09 | 0.363 | 6.1% |  |
| Hysterectomy type | Hysterectomy (unclassified) | | 4.83 (2.10, 7.55) | 3 | 14.11 | 0.000 | 95.0% | 0.002 |
|  | Abdominal hysterectomy | | 1.03 (0.26, 1.80) | 1 | 0 | 0.519 | 0.0% |  |
|  | Laparoscopic hysterectomy | | 4.02 (1.96, 6.09) | 1 | 0 | 0.575 | 0.0% |  |
| Control group | Similar age | | 2.46 (0.89, 4.03) | 2 | 2.52 | 0.001 | 76.3% | 0.087 |
|  | Myomectomy | | 5.45 (2.41, 8.48) | 3 | 15.09 | 0.000 | 94.2% |  |
| All studies | |  | 4.07 (1.78, 6.37) | 5 | 0 | 0.000 | 95.5% | - |

Annotation: BMI=body mass index.
