# Supplementary figures and images for: Effect of hysterectomy on ovarian function: a systematic review and meta-analysis
Source: J Ovarian Res. 2023 Feb 9;16:35. doi: 10.1186/s13048-023-01117-1 (PMC9912518; doi:10.1186/s13048-023-01117-1)

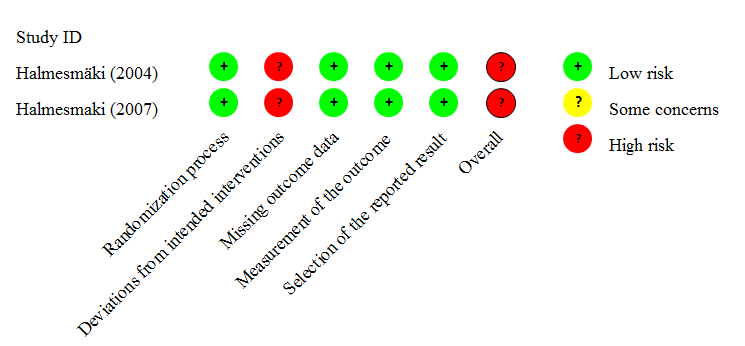

Supplement: Supplementary file 8 — Additional file 8: Figure S1. Bias risk summary for the 2 included RCTs based onthe RoB 2 tools. [file 13048_2023_1117_MOESM8_ESM.tif]

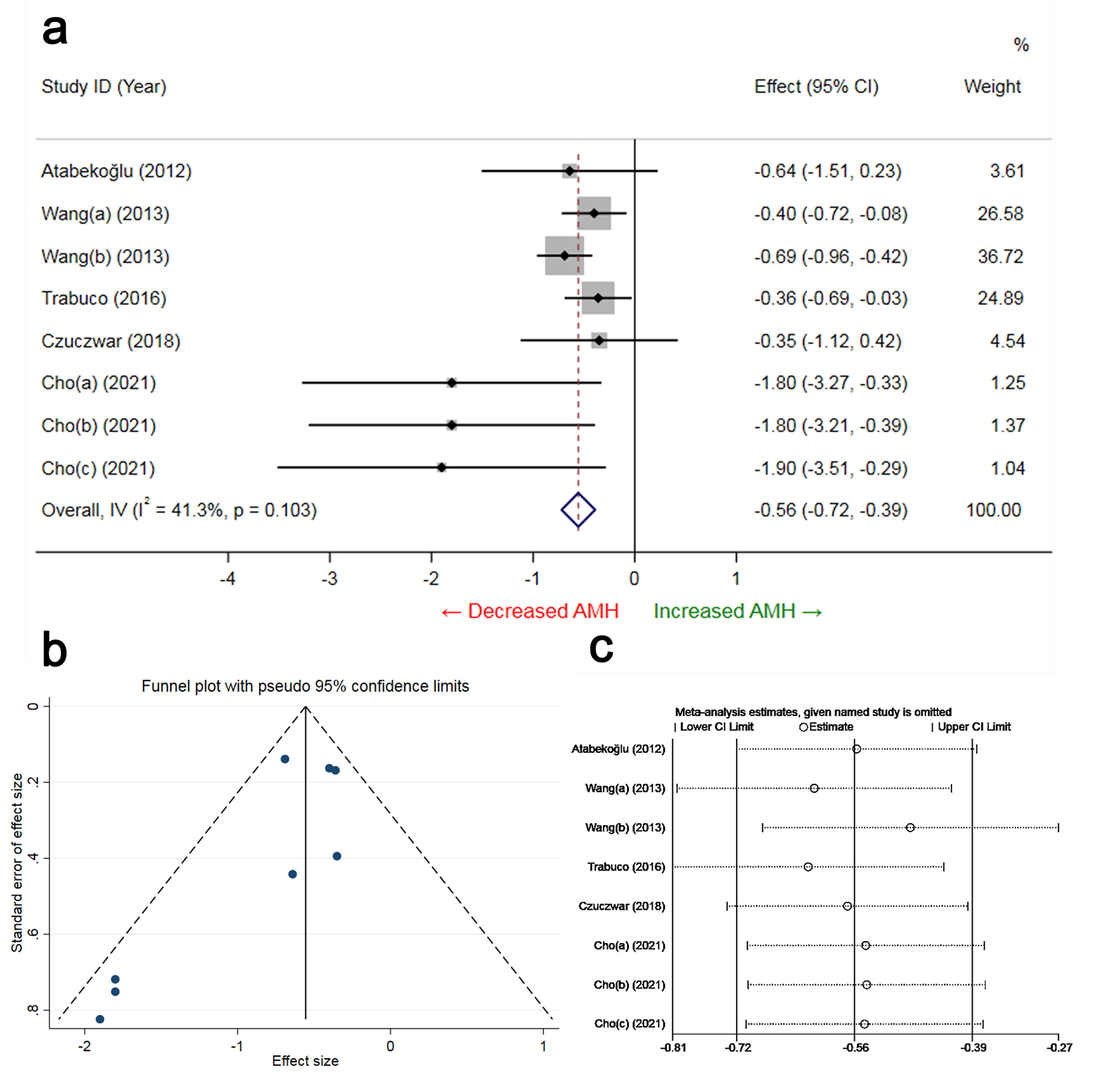

Supplement: Supplementary file 9 — Additional file 9: Figure S2. (a) Forest plot of overall WMD for AMH amongwomen underwent hysterectomy; (b) Funnel plot for assessing publication biaswithin studies related to AMH; (c) Sensitivity analysis for studies related toAMH. [file 13048_2023_1117_MOESM9_ESM.tif]

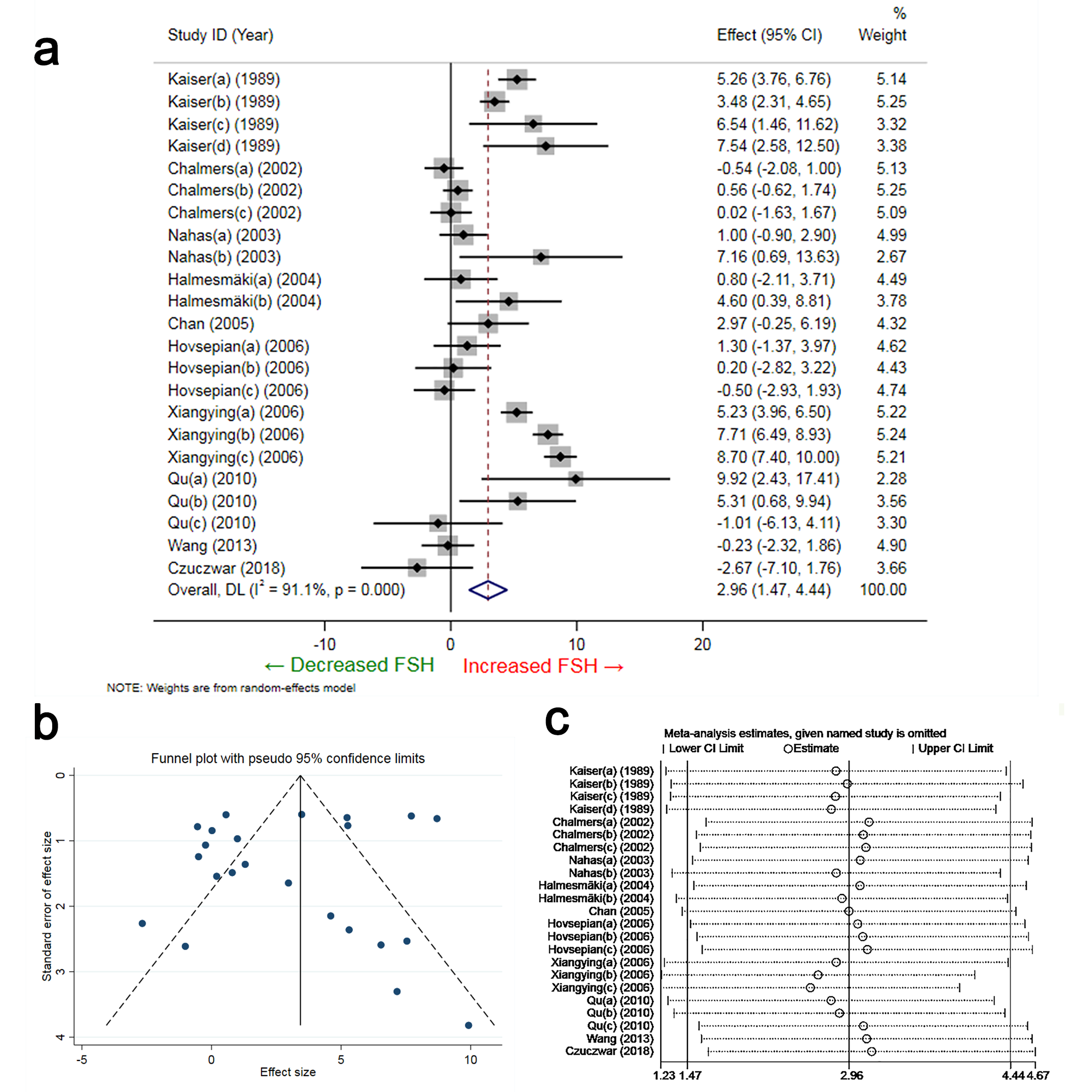

Supplement: Supplementary file 10 — Additional file 10: Figure S3. (a) Forest plot of overall WMD for FSH amongwomen underwent hysterectomy; (b) Funnel plot for assessing publication biaswithin studies related to FSH; (c) Sensitivity analysis for studies related toFSH. [file 13048_2023_1117_MOESM10_ESM.tif]

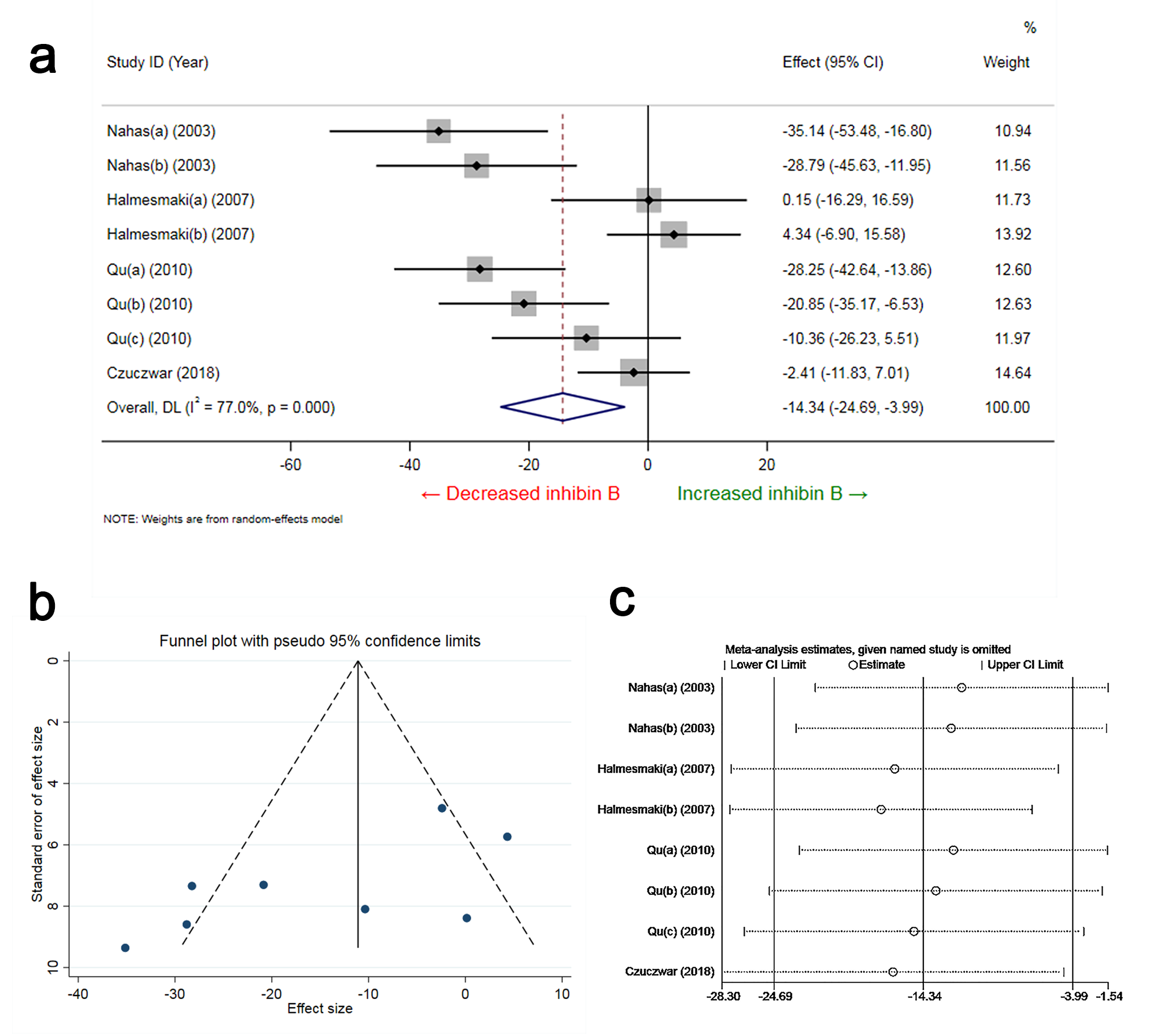

Supplement: Supplementary file 11 — Additional file 11: Figure S4. (a) Forest plot of overall WMD for inhibin Bamong women underwent hysterectomy; (b) Funnel plots for assessing publicationbias within studies related to inhibin B; (c) Sensitivity analysis for studiesrelated to inhibin B. [file 13048_2023_1117_MOESM11_ESM.tif]

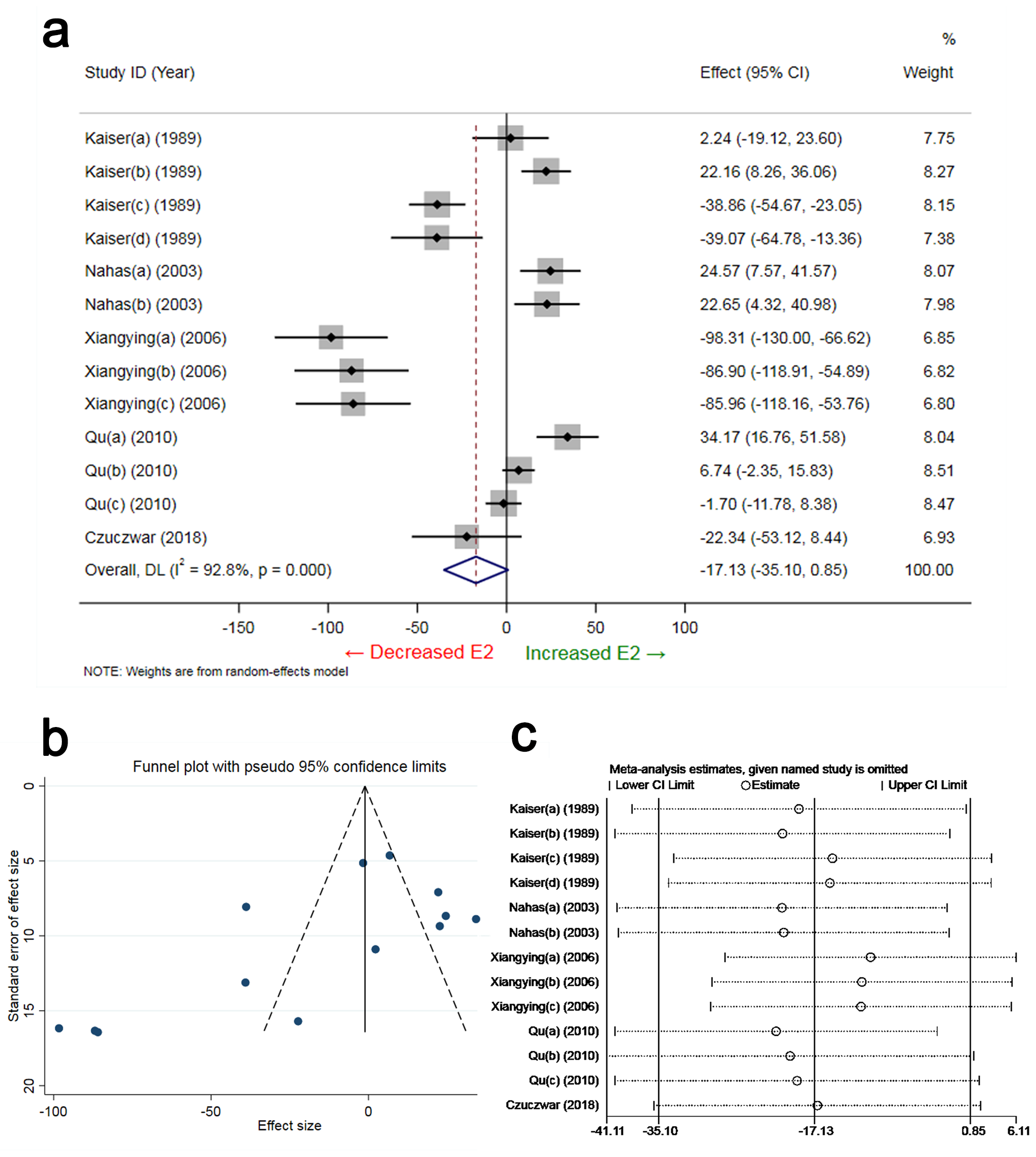

Supplement: Supplementary file 12 — Additional file 12: Figure S5. (a) Forest plot of overall WMD for E2 amongwomen underwent hysterectomy; (b) Funnel plot for assessing publication biaswithin studies related to E2; (c) Sensitivity analysis for studies related toE2. [file 13048_2023_1117_MOESM12_ESM.tif]

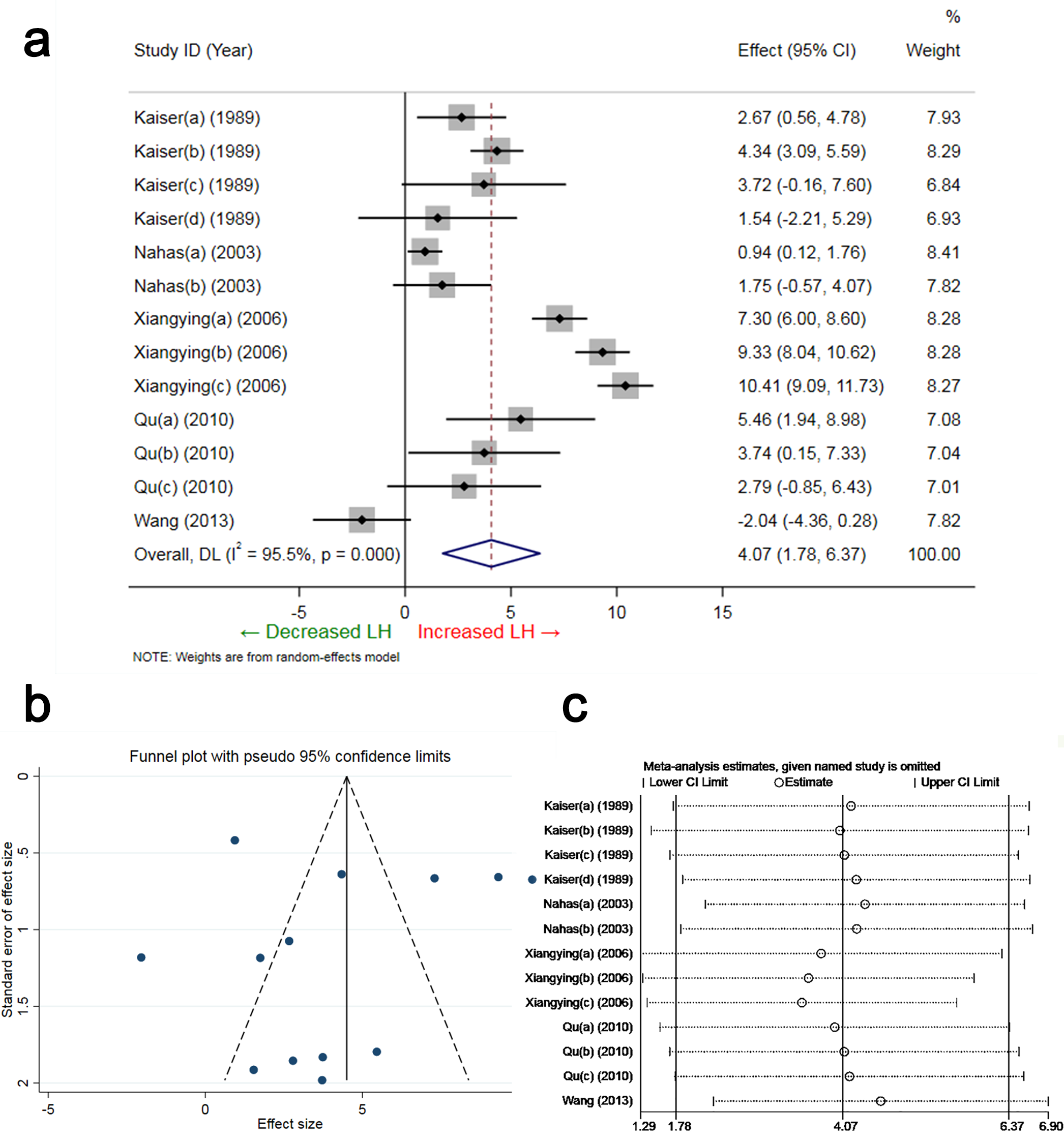

Supplement: Supplementary file 13 — Additional file 13: Figure S6. (a) Forest plot of overall WMD for LH amongwomen underwent hysterectomy; (b) Funnel plot for assessing publication biaswithin studies related to LH; (c) Sensitivity analysis for studies related toLH. [file 13048_2023_1117_MOESM13_ESM.tif]
